# The volatile anesthetic isoflurane causes global suppression of neuronal activity, disrupting hub neuron function in *Caenorhabditis elegans*

**DOI:** 10.3389/fnsys.2026.1795887

**Published:** 2026-06-12

**Authors:** Andrew S. Chang, Laura Mazuera, Christopher W. Connor, Christopher V. Gabel

**Affiliations:** 1 Department of Pharmacology, Physiology and Biophysics, Chobanian and Avedisian School of Medicine, Boston University, Boston, MA, United States; 2 Department of Anesthesiology, Mass General Brigham, Boston, MA, United States; 3 Neurophotonics Center, Boston University, Boston, MA, United States

**Keywords:** anesthesia, *C. elegans*, hub neurons, isoflurane, neuronal imaging

## Abstract

**Introduction:**

Volatile anesthetics, such as isoflurane, generate a state of unconsciousness and analgesia across the animal kingdom and are widely used in clinical settings. Yet, anesthetic mechanisms are poorly understood: the volatile anesthetics are so profligate in their potential effects that it has proven difficult to determine which actions are most causal at the systems level.

**Methods:**

To test if specific cellular targets mediate the anesthetic effect across a complete, intact nervous system, we imaged neuron activity in the *Caenorhabditis elegans* head ganglia at cellular resolution. We measured the effect of increasing anesthetic concentrations across a range of identified neurons within the *C. elegans* nervous system.

**Results:**

However, rather than dramatic effects on any particular neuronal class, we measured uniform suppression of both neuron activity and connectivity with increasing isoflurane across the nervous system. We find the degree of activity suppression to be proportional to the baseline activity of the neuron in the awake state. Within this context, highly connected neurons, specifically neurons with high in-degree connectivity, are inherently active and display large activity suppression. These include hub interneurons within the *C. elegans* command locomotory circuit that control behavioral crawling states and contribute to system-wide coherence of neuron dynamics. By analyzing the effect of isoflurane on the activity of two specific hub interneuron classes, AVA and AVE, we show that the large degree of suppression observed in these neurons corresponds to high baseline activity.

**Discussion:**

Exploiting the small size, simplicity and optical accessibility of *C. elegans*, our results demonstrate that isoflurane anesthesia globally suppresses activity and connectivity across a wide range of neuron types, and suggest a model of anesthesia in which proportional suppression of activity results in disruption of highly connected, highly active, hub loci that are critical to nervous system coordination and state dynamics.

## Introduction

Volatile anesthetics are inhaled drugs that produce a reversible state of unconsciousness and analgesia in organisms spanning the animal kingdom. Despite their ubiquitous clinical usage, no consensus exists on the molecular and cellular action of these drugs. Volatile anesthetics are profligate, with evidence implicating many putative postsynaptic and presynaptic targets. These include agonism of inhibitory GABA_A_ and glycine receptors (Mascia et al., 2000; Mihic et al., 1997; Krasowski and Harrison, 2000), inhibition of excitatory NMDA glutamate (Martin et al., 1995; Solt et al., 2006) and nicotinic acetylcholine receptors (Flood et al., 1997; Xu et al., 2000) as well as disruption of presynaptic neurotransmitter release (Maclver et al., 1996; Perouansky et al., 1995; van Swinderen et al., 1999; Troup et al., 2019; Zalucki et al., 2015). Defects in mitochondrial complex I produce volatile anesthetic hypersensitivity in nematodes, mice and humans (Kayser et al., 2001; Quintana et al., 2012; Zimin et al., 2016), while halogenated ethers have been shown to modulate inhibitory leak current through K^+^ channels (Heurteaux et al., 2004; Patel et al., 1999). But what are the causal actions of volatile anesthetics across an intact nervous system and, more specifically, do certain neuron-types mediate the effects? These questions remain critical to understanding the effects of volatile anesthetics on nervous system function at the network level.

The mechanisms of volatile anesthetics can be studied through their functional effects on neuronal dynamics and network connectivity. While medical EEG monitoring provides a quantifiable measurement of brain activity associated with anesthesia, the EEG has limited ability to reveal functional changes at the level of individual neurons and simple circuits. By contrast, modern neuro-imaging techniques such as fMRI and fluorescence microscopy, as well as higher resolution and multi-neuron electrical recordings, coupled with quantitative network analyses, have begun to provide a system-wide understanding of the functional effects of volatile anesthetics. Broadly, the anesthetized state displays an overall suppression in network efficiency and a breakdown in information transfer between network nodes, or “hubs” (Mashour, 2017). Intracortical recordings in mice under desflurane anesthesia reveal a breakdown of global functional connectivity resulting from accumulated disruption in local circuits (Hudetz et al., 2020). Our lab has performed similar analyses in *C. elegans* using single-neuron resolution calcium imaging, showing that the anesthetized state is characterized by a collapse of system-wide functional organization as measured by neuron pair correlations (Awal et al., 2020; Awal et al., 2018).

The nematode worm *C. elegans* is an excellent model system for the study of the basic mechanisms of anesthesia given its simple, completely mapped neuro-connectome (Cook et al., 2019), capabilities for comprehensive multi-neuron functional imaging, and behavioral and neurological responses to volatile anesthetics that parallel higher organisms. Its nervous system holds many highly conserved neurotransmitters in common with humans, including glutamate, GABA, and acetylcholine, as well as homologous receptors (Hobert, 2013; Luersen et al., 2016). As with nervous systems across the animal kingdom, its connectome exhibits “rich club” architecture, possessing a cohort of highly-connected hub neurons which are key to maintaining system-wide coherence and function (Towlson et al., 2013; Uzel et al., 2022).

Each of 302 neurons in the hermaphrodite *C. elegans* has been characterized anatomically, uniquely named, and pan-neuronal gene expression and neurotransmission properties mapped (Taylor et al., 2021; Gendrel et al., 2016; Pereira et al., 2015; Serrano-Saiz et al., 2013). The animal’s small size and transparency allow for volumetric calcium imaging of neuron activity across the entire head of the animal with single cell resolution. The NeuroPAL fluorescent marker system allows for identification of individual neurons within the animals completely mapped neuro-connectome through a combination of differentially expressed nuclear fluorophores and standardized anatomic relationships (Yemini et al., 2021). Thus, identifiable neurons within system-wide multi-neuron activity data sets can be assessed based on innate properties such as neurotransmitter, receptor expression, and synaptic connectivity. These capabilities allow us to systematically investigate how isoflurane anesthesia alters activity patterns of individual neurons within an intact nervous system with a degree of fidelity not possible in higher organisms. In this study, we exploit these advantages of *C elegans* to measure the functional effects of isoflurane at the neuron and network level and understand how this leads to the alterations in global network dynamics characteristic of the anesthetized state.

## Materials and methods

### *C. elegans* strains and maintenance

*C. elegans* were raised at 20 °C on nematode growth medium agar seeded with a lawn of OP50 E. coli. All animals used in this study were a cross between two transgenic strains: QW1217; OH15263. The first transgenic strain, QW1217 (*zfIs124[Prgef-1: GCaMP6s]; otIs355[Prab-3: NLS:tagRFP]*), expresses nuclear-localized tagRFP, a fluorophore facilitating neuron tracking, and cytoplasmic GCaMP6s, a fluorescent calcium reporter facilitating capture of neuronal activity (Chen et al., 2013). The second transgenic strain is OH15263 (*otIs670*), which expresses the NeuroPAL genetic cassette (Yemini et al., 2021). The NeuroPAL cassette induces distinct patterns of expression of three nuclear-localized fluorophores (mNeptune2.5, CyOFP1, and mTagBFP2) across the *C. elegans* nervous system. The expression of these fluorophores is controlled by a set of promoters, designed to allow neuronal identification to be performed by comparing the relative locations of neurons and relative expression (measured via fluorescence) of the three fluorophores. QW1217 was obtained as a gift from the Alkema Lab (University of Massachusetts Medical School, Worcester, Massachusetts) and OH15263 was obtained from the Caenorhabditis Genetics Center (University of Minnesota, Minneapolis, Minnesota). All *C. elegans* used in this study were young adult hermaphrodites.

### Animal imaging preparation

Animals were paralyzed prior to isoflurane exposure and imaging through immersion in S-basal buffer (100 mM NaCl, 50 mM KPO4 buffer, 5 ug/mL cholesterol) containing 5 mM tetramisole, a selective levamisole receptor agonist. Levamisole receptors are a subtype of nematode-specific nicotinic acetylcholine receptor that, in the *C. elegans,* are largely expressed at the neuromuscular junction of body wall muscles, although some neuronal expression has been reported (Culetto et al., 2004). Tetramisole exposure induces muscle depolarization, causing spastic contraction and eventually rigid paralysis (Martin et al., 2012). At the concentrations used, paralysis occurs within a few minutes. We have previously shown that at the levels used, *C. elegans* can be maintained under tetramisole paralysis for at least 2 h while maintaining consistent neuronal dynamics (Chang et al., 2023). After paralysis, animals were embedded in a transparent polyethylene glycol hydrogel before being subjected to anesthetic exposure and imaging procedures (Burnett et al., 2018).

### Isoflurane anesthesia and imaging procedure

Hydrogel embedded animals were equilibrated to specific atmospheric concentrations of isoflurane to produce stable depths of anesthesia. To induce anesthesia to a given atmospheric percentage of isoflurane, we first equilibrate 50 mL of S-basal buffer to that percentage of isoflurane by exposing the buffer to a surface atmosphere of isoflurane at that percentage in a sealed petri dish for 30 min. The hydrogel embedded animal is then submerged in the pre-equilibrated buffer pool, and the pool and animal then re-exposed to the specified percentage of atmospheric isoflurane for an additional 30 min. All S-basal used in this procedure includes 5 mM tetramisole to maintain muscular paralysis.

We have previously shown that 50% of animals no longer respond to sharp touch when anesthetized to 3% atmospheric isoflurane using this procedure, a level which is analogous to a surgical plane of anesthesia in humans (Awal et al., 2018). Therefore, atmospheric concentrations of 2 and 4% isoflurane were chosen to, respectively, represent light and moderate depths of anesthesia. All animals in this study were subjected to a progressive anesthetization and imaging procedure. After paralysis and hydrogel encapsulation, animals were rested for 30 min, and then imaged for 10 min, generating a baseline “0%” recording. Animals were then exposed to 2% isoflurane for 30 min, followed by 10 min of imaging, then exposed to 4% isoflurane for 30 min, followed by a final 10 min of imaging.

### Light sheet microscopy

**Figure 1:**
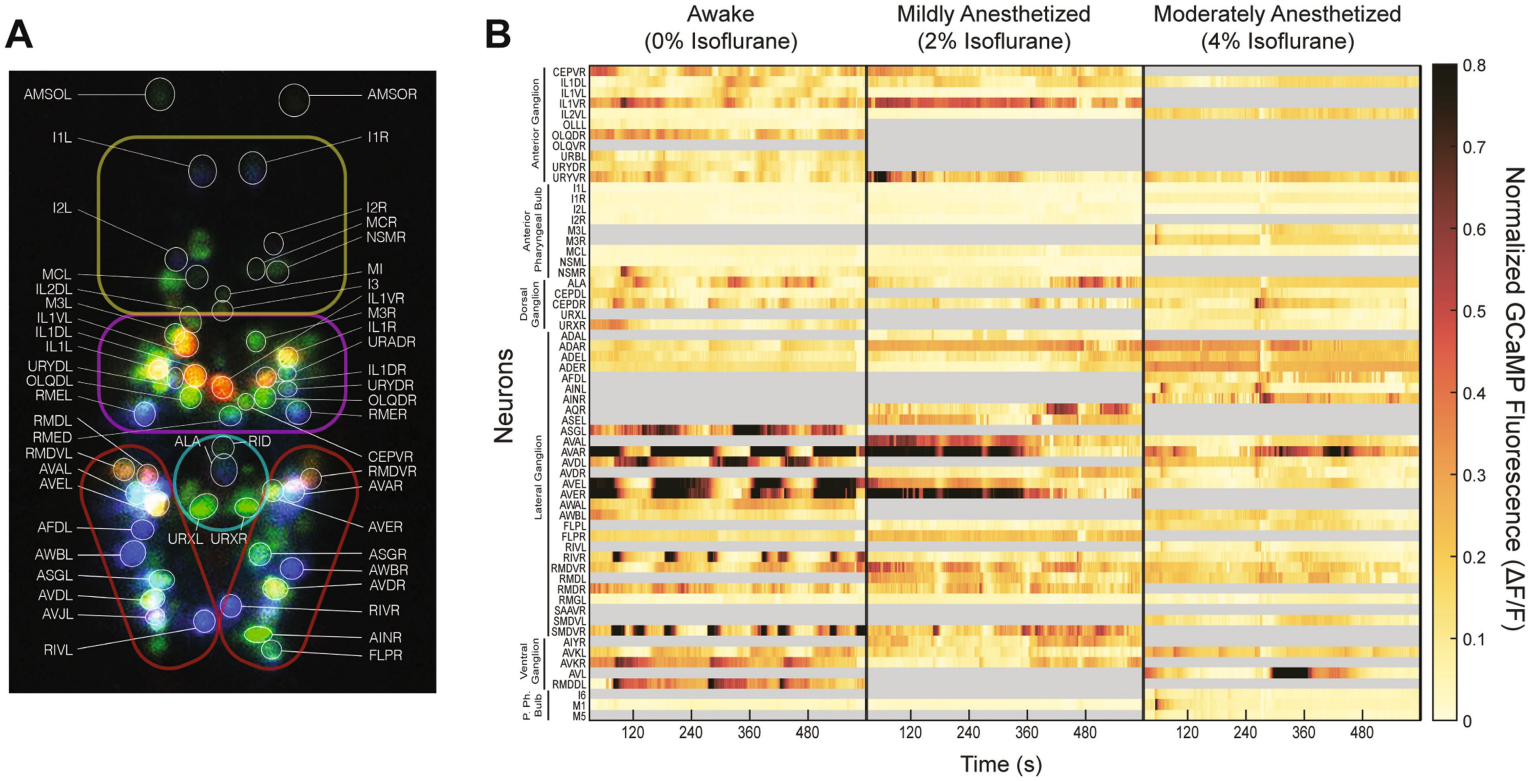
Capture of neuronal dynamics in *C. elegans* at varying depths of isoflurane anesthesia. **(A)** Maximum intensity projection image of the head of a *C. elegans* showing false-color representation of the NeuroPAL fluorescence markers (mTagBFP2 is shown in blue, CyOFP1 is shown in green, and mNeptune2.5 is shown in red) used for neuron identification. Identified neurons are as labeled. The enclosures delineate ganglia including the anterior pharyngeal bulb (yellow); the anterior ganglion (magenta); the dorsal ganglion (turquoise); the lateral ganglia (scarlet). The ventral ganglion, which is located more caudally is not shown. **(B)** Neuronal dynamics as measured by GCaMP6s fluorescence (ΔF/F normalized) captured throughout head ganglia in an individual exemplar animal during three distinct states: awake, mildly anesthetized (equilibrated to 2% atmospheric isoflurane), and moderately anesthetized (equilibrated to 4% atmospheric isoflurane). In between each 10-min recording period, animals were equilibrated to the next greater level of anesthesia for 30 min. Only neurons that were able to be identified via NeuroPAL in at least one recording were shown. Successful identification in one recording did not guarantee identification in any other recording, leading to gaps in data as shown. Citation: Chang et al (2025). License: CC BY. Source: PLoS One.

Volumetric fluorescent microscopy was performed using a Dual Inverted Selective Plane Illumination (diSPIM) fluorescence microscope (Applied Scientific Instrumentation, Eugene, OR) with 0.8 NA 40x water immersion objectives (Nikon USA, Melville, NY; Awal et al., 2020). To capture neuronal activity, tagRFP and GCaMP6s fluorescence were imaged in the head region of each animal (*n =* 15) for three sessions of 10 min each, at 0, 2, and 4% isoflurane concentrations. Volumes were captured at a rate of 2 Hz with a voxel size of 0.1625 × 0.1625 × 1 μm. Before and after each activity imaging session, a single volume capturing tagRFP and the three NeuroPAL fluorophores (mNeptune2.5, CyOFP1, and mTagBFP2) was obtained to facilitate neuronal identification. An example image displaying the maximum intensity projection of a *C. elegans* head with NeuroPAL fluorophores is displayed in Figure 1A.

### Signal extraction and neuron identification

RFP-labeled neuronal nucleus tracking and extraction of GCaMP6s fluorescence signal from neuronal soma was performed as described in previous work from our group (Awal et al., 2020). 150 neuronal activity traces were extracted from each 10-min volumetric recording using custom Python and MATLAB (Mathworks, USA) scripts. Neuron ID was performed using pre and post-imaging NeuroPAL volumes. Neurons in the pre and post imaging volumes corresponding to each 10-min activity imaging session were identified using the NeuroPAL method (Yemini et al., 2021), considering relative anatomical position of neurons and relative brightness of the three NeuroPAL fluorophores in each neuronal nucleus. Neurons in which putative IDs in both pre and post imaging NeuroPAL volumes matched and in which the specified nuclei in both pre and post volumes could be verifiably linked to the same neuron in the corresponding activity imaging session were considered positively IDed.

**Figure 2:**
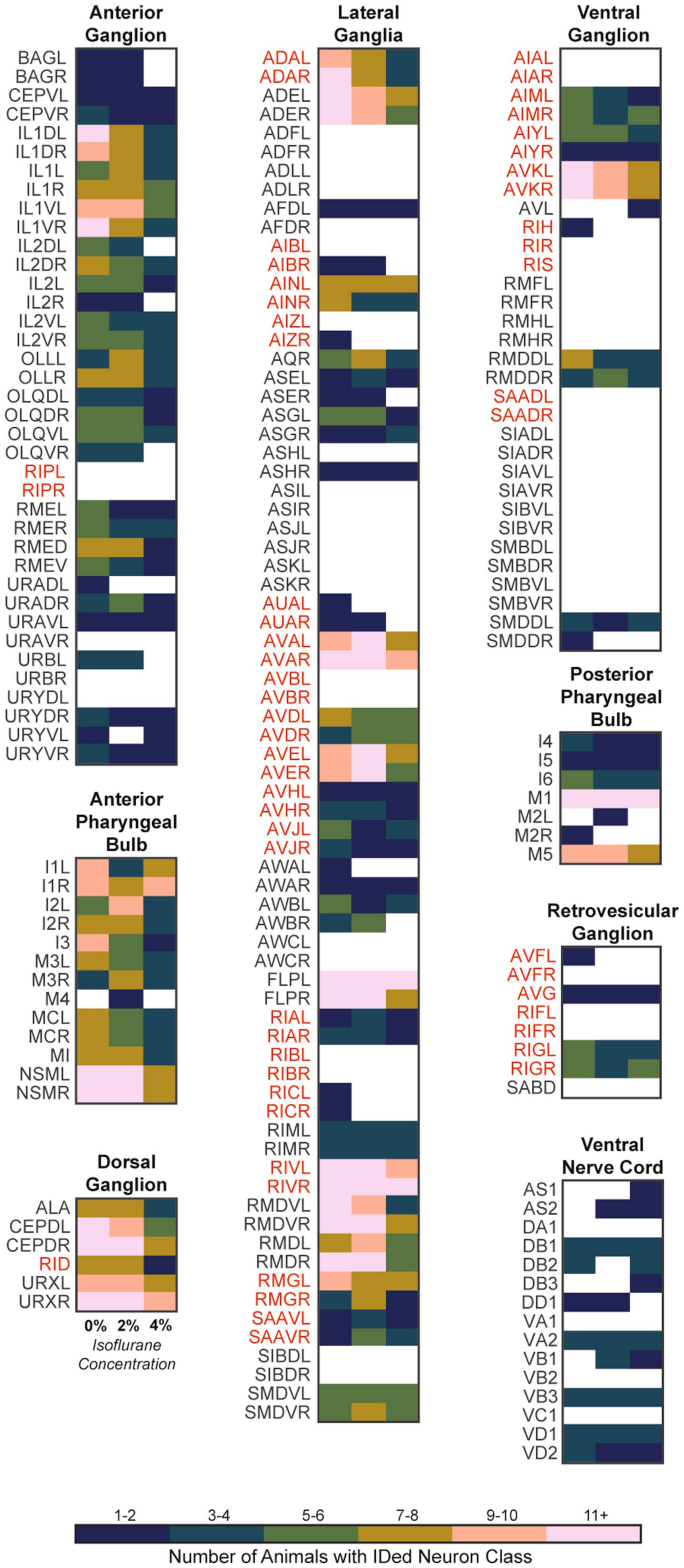
Survey of neurons identified across recording conditions. Color indicates the number of times each neuron class was positively identified in each of the three recording conditions (0, 2, and 4% atmospheric isoflurane). Each neuron class could have been identified as many as 15 times across the sample of 15 animals. A gray entry signifies that a neuron class was never positively identified. Red text labels indicate neurons classified as interneurons within the *C. elegans* connectome.

Out of 45 imaging sessions (*n =* 15 animals at three anesthesia conditions each), an average of 44.6 neurons were positively IDed per session, with a minimum of 21 and a maximum of 69 (excluding one session each at 2% isoflurane 4% isoflurane in which no positive identifications could be made, and one session at 4% in which only one positive identification could be made due to insufficient image quality). A total of *n =* 991 neurons were positively identified at any of the three anesthesia conditions, with *n =* 760/678/448 neurons being identified at the 0%/2%/4% isoflurane conditions. 256 neurons were positively IDed across all three conditions. Figure 1A shows the IDed GCaMP activity traces obtained from a single animal across the three conditions. Because only the head of each animal was imaged, we only considered neurons of the head ganglia and the most anterior neurons of the ventral nerve cord to be possible targets for identification. Figure 2 summarizes the number of times each neuron type in the *C. elegans* head (segregated by ganglion) was positively IDed across all 15 animals. Supplementary Figure S1 may be referenced as a comprehensive survey of neuron identification for the dataset analyzed in this manuscript, providing specification and quantification of which neurons classes were identified in which animals and recording conditions.

### Data analysis

IDed GCaMP6s signal traces were individually normalized before analysis to *Δ*F/F_0_, with F_0_ calculated as the mean value of the lowest 1% of fluorescence observed in each trace. Because fluorophores tend to fluoresce particularly brightly when first illuminated, the first 30 s of signal recorded from each imaging session were excised prior to normalization and were therefore omitted from all subsequent analysis. To assess levels of overall neuronal activity within each IDed signal trace, “signal variance” (SV), or the standard deviation of the normalized signal, were calculated for each trace. Neuronal traces were assorted based on features derived from neuron ID: neurotransmitter identity (Pereira et al., 2015; i.e., “ergic” type) and rich club membership (Towlson et al., 2013; Uzel et al., 2022). *C. elegans* possesses 11 neurons that satisfy the statistical properties of a rich club: they are a set of high-degree neurons which are more highly interconnected than would be expected based on random chance alone. To assess isoflurane induced changes in neuronal activity levels, we assessed the difference in signal variance (Δ signal SD), or “activity shift” for each neuron, between the anesthetized states (2 and 4% isoflurane) and the awake state (0% isoflurane).

To assess isoflurane induced changes in synchrony between synaptically linked neuron-pairs, we generated a list of all pairs of IDed neurons putatively linked by chemical synapses using the hermaphrodite connectome generated by Cook et al. (2019) Because we were primarily interested in drug exposure induced changes, we generated subset populations of neuron-pairs found at both 0 and 2% isoflurane conditions (*n =* 1,418) and at both 0 and 4% isoflurane conditions (*n =* 672). These neuron pairs were then assorted further based on neurotransmitter identity of the presynaptic partner as derived from neuron ID (Pereira et al., 2015). Synchrony between neuron pairs was calculated as the Pearson correlation coefficient (PCC) between the differentiated activity signals of pair members.

To investigate the interaction between neuron connectivity and isoflurane-induced neuronal activity change, we determined the in-degree and out-degree of each neuron type using the hermaphrodite chemical connectome (Cook et al., 2019). In-degree was defined as a neuron’s number of presynaptic partners and out-degree was defined as a neuron’s number of post-synaptic partners.

### Statistical methods

For dataset validation, to quantify how our identified neuron sample differs from the population of all tracked neurons, we applied two-way mixed ANOVA with signal variance as outcome. One-way ANOVA was applied to assess for differences in mean signal variance across animals within each recording condition.

One-way ANOVA was used to compare difference in mean signal variance across anesthesia conditions for different neuronal subgroups. Post-hoc pairwise comparisons were performed using the Tukey–Kramer method. Differences between pre- and post-anesthesia PCC distributions were performed using the Kolmogorov–Smirnov test, and separation of means was quantified using Cohen’s d.

Correlation between neuronal in-degree or out-degree and activity shift was determined using Spearman’s rank correlation coefficient. Comparison of activity shift between rich club neurons and all other neurons was performed using the Wilcoxson rank-sum test. A non-parametric test was chosen because of a large discrepancy between sample sizes of compared populations.

Pre- and post-anesthesia signal variance within individual neuron classes (AVA and AVE) was compared using the paired-sample *t*-test. Correlation between baseline signal variance and isoflurane-induced activity shift was assessed using linear regression.

### Code accessibility

Custom image and data analysis code as well as original imaging data sets are available from the authors upon request.

## Results

### Neuron identification and imaging the response to isoflurane anesthesia

There is evidence that volatile anesthetics interact with a large array of neurotransmitter systems, both postsynaptically and presynaptically (Chau, 2010; Weinrich and Worcester, 2018). However, it remains unclear if there is a selective effect of isoflurane on any distinct class of neurons that drives the functional effects of anesthesia. Employing fluorescence microscopy in *C. elegans,* we can simultaneously image individual neuron activity across many neurons in the animal’s head and identify them within the complete connectome using the NeuroPAL marker system. Figure 1A illustrates an example image of the NeuroPAL fluorescence markers and identified neuron labels within the head of *C. elegans*. Figure 1B illustrates the calcium fluorescence (ΔF/F normalized GCaMP6s) of identified neurons from an individual example animal during 10 min trials of spontaneous activity at progressively higher levels of anesthesia. In order to effectively measure the functional elements that initiate and drive anesthesia (i.e., those that are affected first, most dramatically), we focused our imaging assays over a range of mild to moderate levels of isoflurane [i.e., 0, 2, 4% atmospheric isoflurane, as we determined earlier (Awal et al., 2018)]. Figure 2 illustrates the rate at which each neuron type was successfully identified within our imaging assays, using the NeuroPAL fluorescence markers (see methods) over 15 separate animals. Using this system we successfully identified a majority of neurons in the animals head (63.7% of neurons were IDed at least one time) and identified >30% of neurons in at least five imaging trials. Thus, while identification rates were not comprehensive within individual trials, identified neurons within the complete data set sufficiently cover a broad census of neurons and synaptic subtypes (see Supplementary Figure S1). In particular, we routinely captured numerous interneurons, indicated in red in Figure 2.

**Figure 3:**
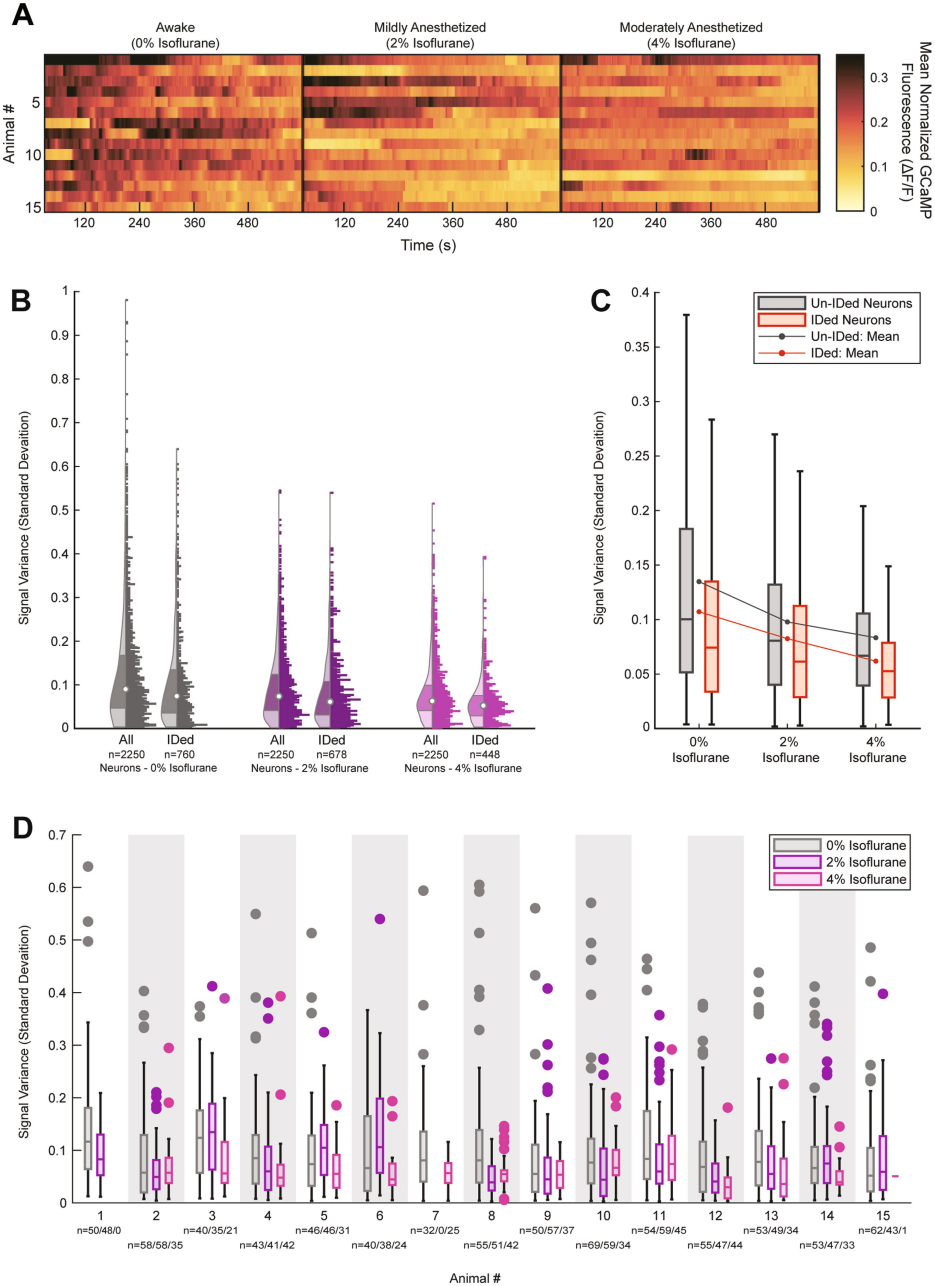
Neuronal activity signal quantified by signal variance across the set of identified and unidentified neurons, and across animals. **(A)** Mean neuronal GCaMP fluorescence signal as recorded from each of *n =* 15 hermaphrodite *C. elegans* across specified depths of anesthesia. Neuronal activity has been averaged across 150 neurons independently tracked from each animal at each depth. **(B)** Violin plots and histograms summarizing distribution of neuronal activity signal as quantified by signal variance (standard deviation of GCaMP fluorescence signal). The distribution of the population of all neurons recorded at each anesthesia recording condition (0, 2, and 4% atmospheric isoflurane) are shown, as well as the distribution of the subset of neurons successfully identified at each condition. White circles show the population median and shaded bars show the interquartile range. **(C)** Comparison of signal variance distribution and means between the population of identified neurons and non-identified neurons at each recording condition. Means are shown by line plots. Boxplots show median and interquartile range (IQR), with whiskers showing non-outlier minima and maxima, with outliers being values > 1.5 times the IQR away from the IQR. Individual outliers have not been plotted. **(D)** Distribution of signal variance across recording conditions and animals. Boxplots show median and interquartile range (IQR), with whiskers showing non-outlier minima and maxima. Individual outlier data points have been plotted. See main text for statistical analysis.

The average calcium fluorescence across all imaged neurons within each animal (*n =* 15) is shown in Figure 3A, with a marked pattern of decreasing fluorescence indicating an increase in neuronal quiescence at greater depths of anesthesia. To further assess the population of neurons we successfully IDed via NeuroPAL compared to the ensemble effects of anesthesia, we compared the distribution of neuronal activity in the set of all tracked neurons to that of the IDed neurons. Neuron activity is quantified by the standard deviation of the ΔF/F normalized GCaMP6s fluorescence signal for each neuron over a given recording block (i.e., the “signal variance”). The distribution and mean across all imaged neurons and the subset of IDed neurons at each condition are shown in Figure 3B. There is a small observable difference in median signal variance (SV), with a lower median SV in the IDed subpopulation compared to the tracked superset in all three anesthesia conditions (*p* values between the mean of all imaged neurons and the IDed subset were 0%: 5 × 10^−6^, 2%: 4 × 10^−4^, 4%: 2 × 10^−11^, via one sample *t*-test). However, SV interquartile ranges are grossly similar when comparing all tracked neurons and IDed neurons across conditions.

As illustrated in Figure 3C, we further compared activity measurements and fluorescence SV, between the subsets of IDed neurons and non-IDed neurons. Although SV distributions are qualitatively largely similar, and while both IDed and non-IDed populations exhibited a dose-dependent depression of SV in response to isoflurane, the IDed population means were consistently lower than the non-IDed population mean (IDed vs. non-IDed, 0%: 0.107 SV vs. 0.135 SV, 2%: 0.083 SV vs. 0.098 SV, 4%: 0.062 SV vs. 0.083 SV). To quantify these differences, we performed two-way mixed ANOVA assessing IDed vs. not IDed and isoflurane condition (0%/2%/4%) as predictive factors for neuronal SV. As expected, depth of anesthesia was significantly predictive for SV, with *p =* 3 × 10^−56^. The IDed vs. non-IDed population was also shown to be predictive for SV with *p =* 2 × 10^−17^, suggesting that the IDed population does differ significantly from the non-IDed population in terms of SV. However, interaction between the factors of being IDed and anesthesia depth was not significant at *p =* 0.085, which crucially suggests that the rate of SV change in response to anesthetic is consistent across the IDed and non-IDed populations. This can be visually observed in Figure 3C, in which the lines depicting the change in SV from 0 to 2% isoflurane and 2 to 4% isoflurane are largely parallel between the IDed and non-IDed populations. Thus while the sample of IDed neurons is not akin to an ideal random sampling from the overall set of tracked neurons, the population of IDed and non-IDed neurons react to isoflurane in a similar way in terms of SV. Therefore, analysis of the metric of SV in response to isoflurane exposure in the IDed subpopulation of neurons should be representative of SV response to isoflurane in neurons in *C. elegans* overall.

To further compare the response across individual animals, we plotted separately the distribution of SV in IDed neurons of all 15 animals and 3 recording conditions in Figure 3D. While the neuron populations at 0% isoflurane exhibit comparatively large numbers of highly active outlier neurons, this behavior is consistent across all 15 animals. Indeed, one-way ANOVA comparing the mean SV across all 15 animals at the 0% isoflurane recording condition does not reject the null hypothesis that means are equal, with *p =* 0.097. The mean SV across animals does appear to differ at the 2% (*p =* 3 × 10^−11^) and 4% (*p =* 3 × 10^−4^) isoflurane conditions. However, some variation between animals in response to isoflurane is expected and consistent with previous observations. While some animals clearly differ from others in terms of distribution, given the largely overlapping interquartile ranges and similar counts of IDed neurons across animals, there do not appear to be gross outlier effects from any specific animal or few animals.

### The response of neurotransmitter subpopulations to isoflurane anesthesia

**Figure 4:**
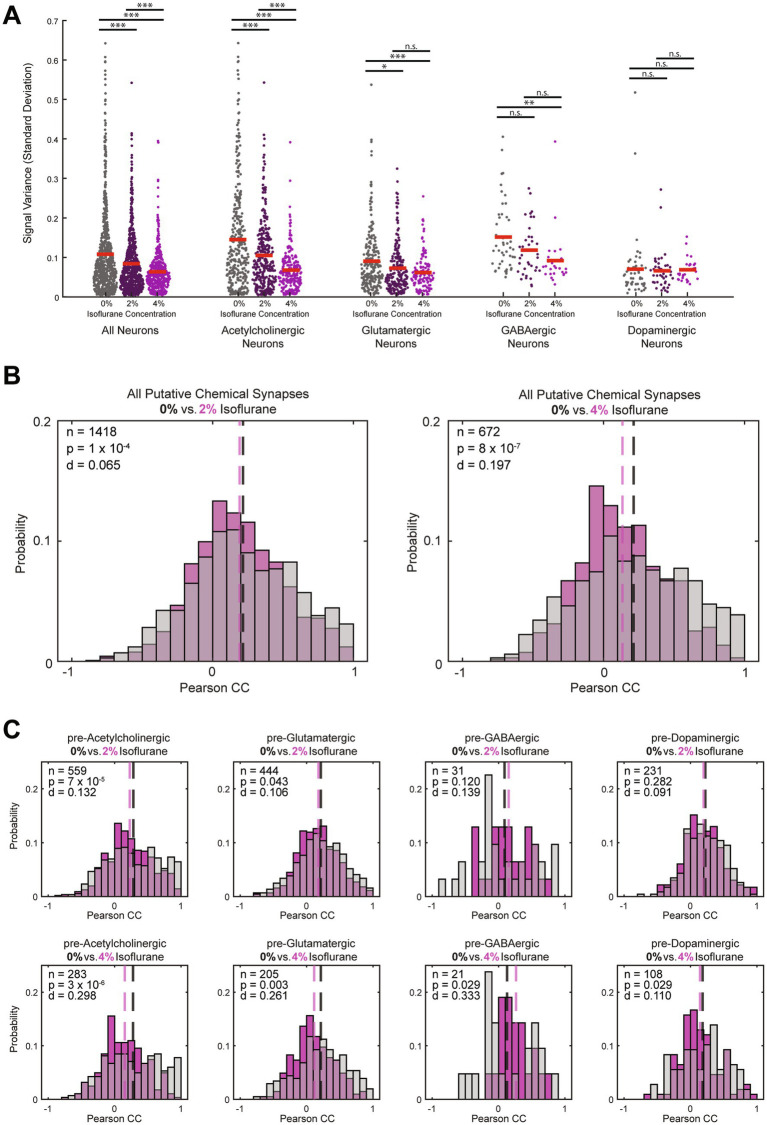
Suppression of neuronal activity and synchrony of distinct neuron populations under isoflurane anesthesia. **(A)** Neuronal signal variance measured as standard deviation of ΔF/F normalized calcium signal. Included are all positively identified neurons recorded from *n =* 15 animals, sorted by neurotransmitter identity and level of anesthesia (*n =* 760/678/448 neurons at 0%/2%/4% isoflurane). Red bars mark population means. Difference of means across anesthesia conditions for each subgroup of neurons were compared using one-way ANOVA, and post-hoc pairwise comparisons were performed using the Tukey–Kramer method. See Table 1 for statistical detail. * *p <* 0.05; ** *p <* 0.01; *** *p <* 0.001 **(B)** Distribution of signal synchrony of neuron pairs connected by chemical synapses as measured by Pearson correlation coefficient (PCC). Only neuron pairs that were positively identified in both the awake condition and in the respective comparison condition (either 2% or 4% isoflurane) were included. Correlation distributions from neuron pairs drawn from awake animals (grey histograms) were compared against those drawn from animals equilibrated 2 and 4% isoflurane (purple histograms) using the Kolmogorov–Smirnov test, and separation between distribution means was measured using Cohen’s d. **(C)** As in **(B)**, but neuron pairs have additionally been sorted by the neurotransmitter identity of the presynaptic partner.

Leveraging the cell-specific information of the *C. elegans* connectome, we can categorize the identified neurons in our imaging data sets by either their presynaptic or postsynaptic properties and assess how neurotransmitter identity corresponds to the degree to which that neuron’s activity is altered in the anesthetized state. As in higher organisms, the majority of neurons in *C. elegans* only express the molecular machinery required to produce one neurotransmitter type (Pereira et al., 2015). This establishes a clean division of the captured neurons into largely mutually exclusive groups. Figure 4A and Table 1 show how different subpopulations of neurons defined by neurotransmitter identity behave under isoflurane anesthesia. As shown in Figure 4A, left, we observe that the dynamic range of neuronal activity, measured by signal variance (SV), becomes suppressed with progressively deeper planes of anesthesia when measured across all identified neurons. These results are similar to that of ensemble measurements for all tracked neurons in the data set (SV for all neurons including those tracked and measured but not identified: 0.125 SV at 0% isoflurane, 0.093 SV at 2% isoflurane, and 0.079 at 4% isoflurane) suggesting that our IDed neuron data set accurately represents the effects of isoflurane on activity across the entire nervous system. These results also recapitulate our previous findings measured across all tracked neurons in the *C. elegans* head (Awal et al., 2020). Similar observations have been documented in mammals in which isoflurane anesthesia is accompanied by overall reduction in activity (Ying et al., 2009). Comparing neuronal subpopulations based on neurotransmitter type, we find that acetylcholinergic neurons exhibit potent isoflurane-induced suppression of activity, with a 28% drop in signal variance (SV) at 2% (0.103 SV) isoflurane and a 55% drop at 4% (0.064 SV) isoflurane compared to controls (0% 0.142 SV). Glutamatergic neurons exhibit a 20% drop in SV at 2% isoflurane (0.089 to 0.071 SV) and a 31% drop SV at 4% isoflurane (to 0.061 SV). GABAergic neurons exhibit a 22% drop in SV from 0% (0.152) to 2% (0.118) isoflurane (although this is not statistically significant by ANOVA analysis) and a 40% drop at 4% isoflurane (0.091 SV). Strikingly, dopaminergic neurons, which are sensory and show low baseline activity levels at 0% isoflurane, exhibits no significant change in SV across all depths of anesthesia.

**Table 1:** ANOVA results, neuronal signal variance across depths of anesthesia for neuronal populations defined by neurotransmitter expression.

One-way ANOVA	Tukey–Kramer *post-hoc*		
All Neurons	0%	2%	*p =* 3 × 10^−7^***
*p =* 1 × 10^−17^*** n = 760/678/448	0%	4%	*p =* 5 × 10^−18^***
	2%	4%	*p =* 3 × 10^−4^***
Acetylcholinergic	0%	2%	*p =* 2 × 10^−6^***
*p =* 2 × 10^−17^*** n = 335/315/223	0%	4%	*p =* 9 × 10^−19^***
	2%	4%	*p =* 5 × 10^−5^***
Glutamatergic	0%	2%	*p =* 0.012*
*p =* 3 × 10^−4^*** n = 235/213/126	0%	4%	*p =* 4 × 10^−4^***
	2%	4%	*p =* 0.375
GABAergic	0%	2%	*p =* 0.131
*p =* 0.006** n = 46/36/26	0%	4%	*p =* 0.005**
	2%	4%	*p =* 0.336
Dopaminergic			
*p =* 0.939 *n =* 52/43/28			
		

Difference of means across anesthesia conditions for each subgroup of neurons were compared using one-way ANOVA, and *post-hoc* pairwise comparisons were performed using the Tukey–Kramer method. See Figure 4A for visualization. *p < 0.05; **p < 0.01; ***p < 0.001.

### Signal coherence of acetylcholinegeric and glutamaterigic synaptic pairs are most sensitive to isoflurane

While the suppression of neuronal activity appears to be a key feature of anesthesia, the anesthetized state is also characterized by a disruption in neuronal connectivity. Our previous work captured isoflurane induced general neuronal discoherence at the cellular level in *C. elegans*. We measured a reduction in the mean activity correlation between all pairs of imaged neurons within the nervous system as a global network metric (Awal et al., 2020), as well as suppression of other metrics such as mutual information and transfer entropy (Awal et al., 2018; Chang et al., 2023). Within our identified neuron data set we first measure activity correlations calculated using the Pearson correlation coefficient (PCC) across all identified synaptic pairs (i.e., the activity correlation between all pairs of identified neurons that are known to be connected via a direct synapse within the connectome). As might be expected, these synaptic pairs show greater correlation than the mean correlation across all pairs of imaged neurons without regard to synaptic connectivity: the mean correlation across all possible pairs of tracked neurons at 0% Isoflurane is 0.0766, which is substantially lower than that of identified synaptic pairs at 0.216. As shown in Figure 4B, with this correlation metric, we measure a mild decrease in the synaptic correlations between 0 and 2% (mean PCC, 0.216 to 0.192) and a larger decrease between 0 and 4% (mean PCC, 0.214 to 0.135) across all identified synapses. Shifts in correlation distributions are quantified by the mean correlation and tested for significance using the Kolmogorov–Smirnov test. These effects recapitulate our previous findings of decreased correlation across the ensemble of tracked but non-identified neurons in the animal’s head (Awal et al., 2020).

The identified dataset allows for further refinement based on individual synapse type. Applying the *C. elegans* chemical connectome to our identified neurons, we categorized sets of neuron pairs that are known to be linked via particular chemical synapses. Thus, the PCC can be calculated for any given subpopulation of synapses to determine how that select distribution is altered in the anesthetized state. For example, we were able to extract *n =* 559 neuron pairs that satisfied the following conditions: (1) putatively connected by a chemical synapse, (2) acetylcholinergic presynaptic partner, (3) both members of the neuron pair were identified in both the 0 and 2% isoflurane trials for that animal. Figure 4C displays the probability distribution histograms of neuron correlations for all ID synaptic pairs of the type and condition indicated. Acetylcholinergic and glutamatergic synaptic pairs at 2% isoflurane exhibit a significant suppression of PCC compared to baseline, with acetylcholinergic pairs decreasing 19% (mean PCC, 0.281 to 0.227) and glutamatergic synaptic pairs decreasing by 17% (mean PCC, 0.215 to 0.178). The effects become larger at 4% with an overall 45% decrease (mean PCC, 0.277 to 0.151) in correlation of acetylcholinergic pairs and a 46% decrease (mean PCC, 0.213 to 0.115) in glutamatergic synaptic pairs. We measured no such significance in the GABAergic pairs at 2%, although it should be noted the limited number of physical GABAergic synapses in the *C. elegans* nervous system (i.e., resulting in low n in the subclass) restrict the statistical power of this measurement. At 4%, GABAergic pairs show a significant increase in correlation (of 51%, mean PCC, 0.128 to 0.262) consistent with a loss of inhibitory/anti-correlated signaling. Dopaminergic pairs show no detectable change at 2% isoflurane and a smaller but significant decrease of 22% (mean PCC, 0.182 to 0.143) at 4%. These results suggest that acetylcholinergic and glutamatergic synaptic pairs experience disruption in synchrony at the earlier 2% isoflurane plane of anesthesia, and that they may drive the overall initial global dyssynchrony we observe across the entire system. By the time the moderate plane of 4% isoflurane is reached, the *C. elegans* nervous system experiences a more uniform disruption in signal coherence across all synapse types.

### Neuron in-degree strongly correlates with isoflurane-induced neuronal activity suppression

**Figure 5:**
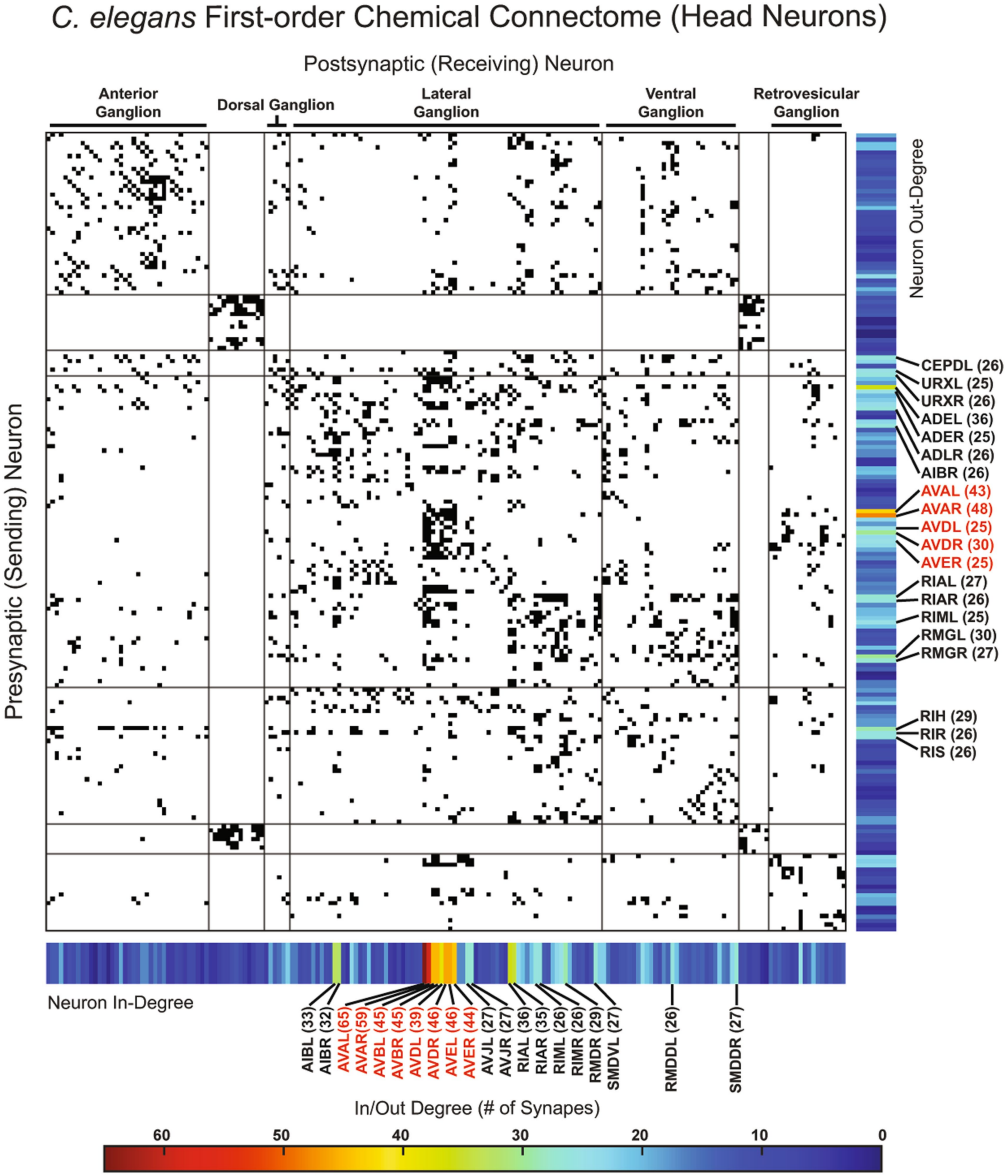
First-order chemical connectome of head neurons in *C. elegans*. Connectivity matrix of neurons found in the *C. elegans* head ganglia. All chemical synapses that are known to exist between pairs of head ganglia neurons are marked. Matrix is derived from the hermaphrodite chemical synapse connectome as described in Cook et al., 2019. In-degree (# of presynaptic partners for a given receptive neuron) and out-degree (# of postsynaptic partners for a given signaling neuron) of each neuron is shown. The in-degree and out-degree of the 20 most prolific receivers and senders are shown. In and out-degrees as reported in parentheses (and as implemented in the remainder of this study) include connections to and from neurons outside of the head ganglia, and which are therefore not displayed in this visual representation of the connectome. “Rich Club” member neurons are marked in red (Towlson et al., 2013).

To what extent do the functional effects of isoflurane depend on the total connectivity of individual neurons, i.e., their overall connectedness, or in- and out-degree? The out-degree of a neuron refers to the number of postsynaptic partners it has: i.e., how many neurons it sends information to. Conversely, the in-degree of a neuron refers to the number of presynaptic partners that neuron has: i.e., how many other neurons it receives information from. Figure 5 visualizes how out- and in-degree can be calculated from the first-order chemical connectome and highlights the highest degree neurons in the *C. elegans* head ganglia. Neurons that have both high out- and in-degree have been described as “hub” neurons, including so called “rich club” neurons that are highly connected among themselves, and are theorized to act as informational “funnels” serving as integrative centers of neuronal signaling (Bonifazi et al., 2009; Gal et al., 2021; Towlson et al., 2013). In *C. elegans*, rich club neurons include four symmetric pairs of the key locomotory interneurons of the lateral ganglia that are responsible for controlling the behavioral state (i.e., forward vs. backward movement etc.) of the animal: AVAR, AVAL, AVBR, AVBL, AVER, AVEL, AVDR, and AVDL (Towlson et al., 2013). Of these we routinely identified AVA and AVE neuron types in our imaging assays, whereas AVD was identified at a lower rate. We were unable to identify the neuron AVB as it is distinguished only by an absence of specific coloration in the NeuroPAL labeling system.

**Figure 6:**
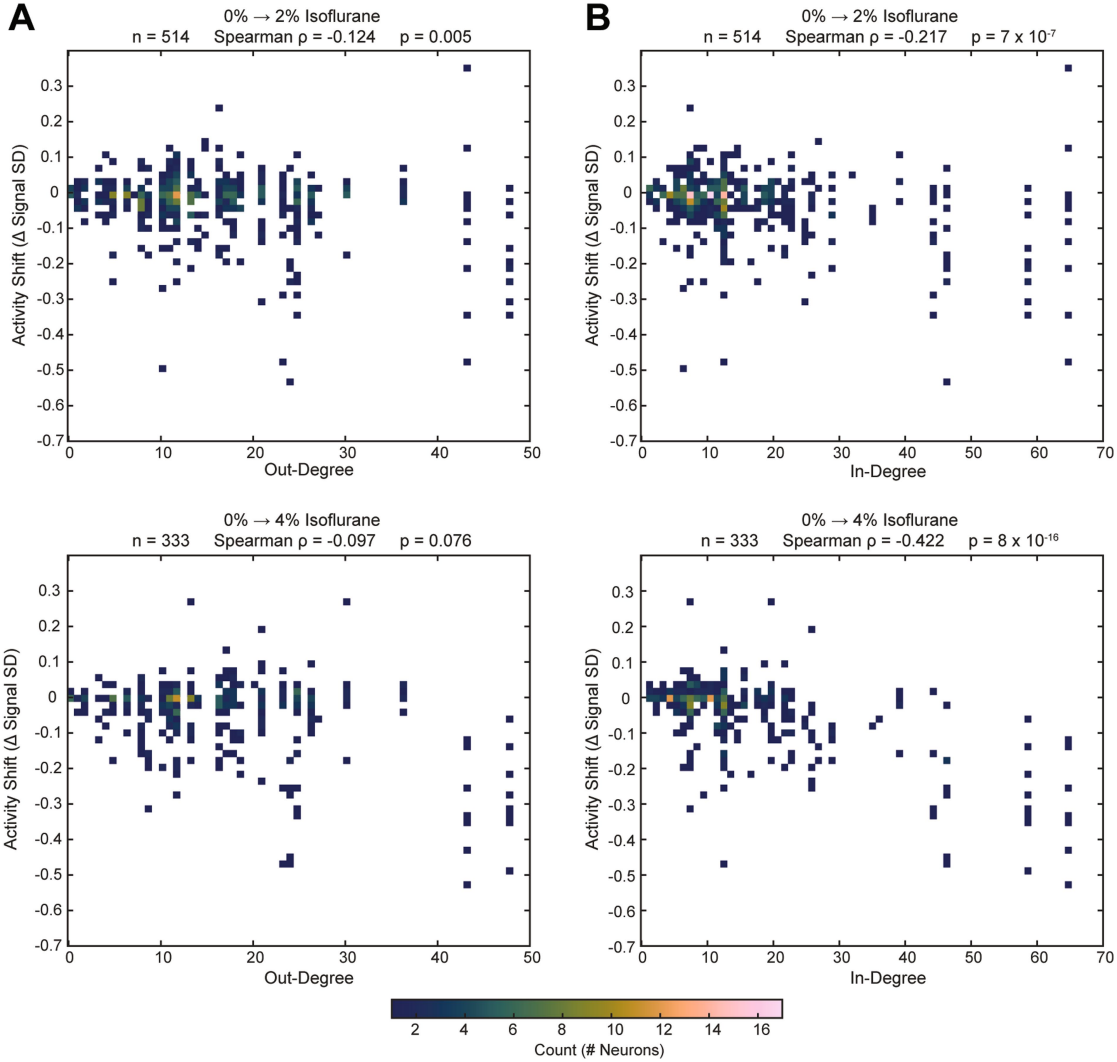
Magnitude of isoflurane induced neuronal activity suppression correlates with increasing in-degree. Activity shift is defined as the difference between the baseline (0% isoflurane) and anesthetized (2% or 4% isoflurane) signal SD of a given neuron. The isoflurane induced neuronal activity shift of each neuron is plotted against the **(A)** out-degree and **(B)** in-degree of the neuron. Correlation between activity shift and out-degree or in-degree was assessed with Spearman’s rank correlation coefficient.

Does the degree of neuron connectivity correspond to the effects of isoflurane on the cellular level? Figures 6A, B show the change in activity (i.e., the “activity shift”) of identified neurons from 0 to 2% isoflurane and from 0 to 4% isoflurane depending, respectively, on the out-degree and in-degree. Comparing the activity shift vs. either the in-degree or the out-degree of each neuron using Spearman’s rank correlation coefficient, we find that the relevance of out-degree is inconsistent, with a mildly significant negative correlation between activity shift and out-degree (*p =* 0.005) when considering activity shift between 0 and 2% isoflurane, but no significance in effect (*p =* 0.076) at 0 to 4% (Figure 6A). However, the relevance of in-degree was conclusive, exhibiting negative correlations between activity shifts and neuron in-degree in both the 0 to 2% (Figures 6B, *P* = − 0.217, *p =* 7 × 10^−7^) and 0 to 4% (Figures 6B, *P* = − 0.422, *p =* 8 × 10^−16^).

**Figure 7:**
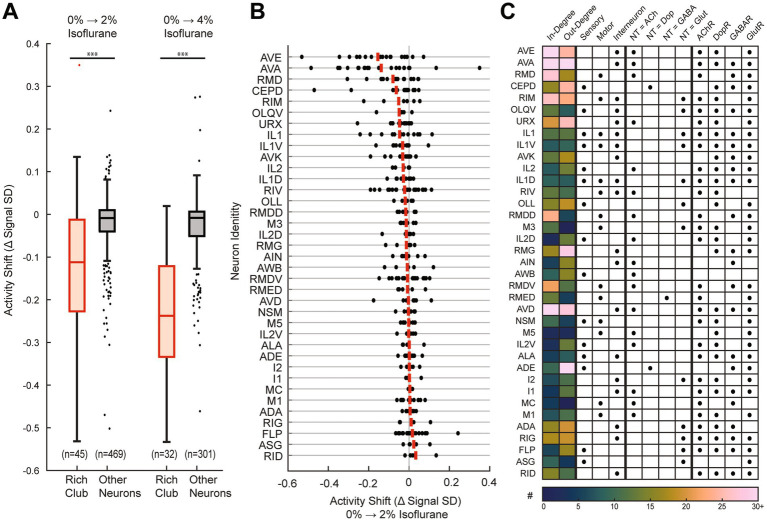
Hub neurons are on average more suppressed than other neurons during isoflurane anesthesia. **(A)** Activity shift of rich club neurons vs. all other identified neurons. Upper and lower box boundaries denote upper and lower quartiles, with central line denoting median. Whiskers denote data range, with the exclusion of outliers, which are defined as being 1.5 interquartile ranges away from the median. Group medians were compared using the Wilcoxson rank-sum test. * *p <* 0.05; ** *p <* 0.01; *** *p <* 0.001. **(B)** Activity shifts of neuron classes that were identified at least 10 times at 0 and 2% isoflurane. Mean shift for each class is marked in red, shaded red indicates 95% confidence interval. Results are sorted from most to least negative mean activity shift (most to least suppressed). Left and right instances of bilateral neuron classes have been pooled. **(C)** In-degree, out-degree, neuron function, neurotransmitter expression, and receptor expression patterns of the neuron classes as sorted in Figure 7B.

We find that within our identified neuron populations, rich club neurons in particular exhibit a large decrease in activity shift after isoflurane exposure in comparison with other neurons (Figure 7A). Further, by grouping the left and right pairs of neurons (the *C. elegans* nervous system is largely bi-laterally symmetric with a high degree of left–right pair symmetry), we can rank frequently identified neuron subtypes by activity shift under mild anesthesia (i.e., 2% isoflurane, Figure 7B). We observed that the rich club neurons AVA and AVE exhibit the greatest activity loss in dynamic range of all measured neuron classes. Both AVA and AVE have high out- and in-degree and are both acetylcholinergic neurons. Figure 7C visualizes the various known properties of these and other significant frequently identified neurons, including neuronal function, neurotransmitter expression, receptor expression as well as in- and out-degree.

### Anesthesia suppression of neuronal activity is proportional to baseline activity

**Figure 8:**
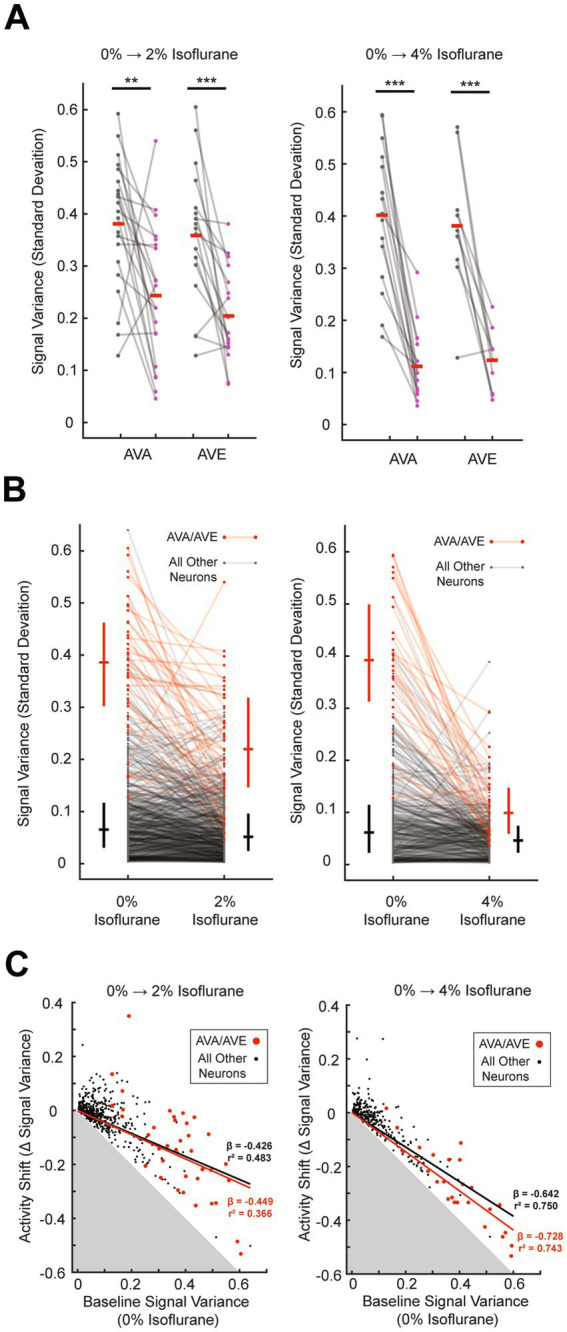
The degree of isoflurane induced suppression is proportional to the neuron’s baseline activity level. **(A)** Paired signal variances measured at baseline (0% isoflurane) and in the anesthetized state (2 and 4% isoflurane) for the populations of the rich club members: AVA and AVE. Group means (red bars) compared using paired-samples *t*-test. * *p <* 0.05; ** *p <* 0.01; *** *p <* 0.001. **(B)** Paired signal variances measured at baseline (0% isoflurane) and in the anesthetized state (2 and 4% isoflurane) for AVE and AVA as well as all other identified neurons. Error bars denote interquartile range and median. **(C)** Activity shift (change in signal variance) for each individual identified neuron from 02 and 04% isoflurane plotted against the neuron’s baseline (0% isoflurane) signal variance. Correlation between baseline signal variance and activity shift assessed using linear regression.

Figure 8A illustrates the change in signal variances of the frequently identified rich club neurons AVA and AVE from 0 to 2% and 0 to 4% isoflurane exposure. Both AVA and AVE exhibit significant suppression of signal variance after 2% isoflurane exposure (36 and 43% of SV respectively). These neuron classes possess extremely similar receptor expression profiles, are both acetylcholinergic, and are remarkably active in the baseline (0% isoflurane) condition. The magnitude of the drop in signal variance induced by isoflurane in AVA and AVE appears to be attributable to the magnitude of the baseline activity levels of these neurons: the bigger they are, the harder they fall. Figure 8B illustrates the change in signal variance of AVA and AVE compared to all other identified neurons (i.e., all neurons that were successfully identified in both conditions in the same animal). At 0% isoflurane the interquartile range for AVA and AVE signal variance exceeds the interquartile range (indicated by the vertical bar) for all other neurons lumped together. The effect of isoflurane is to significantly reduce AVA/AVE signal variance. The remaining neuronal cohort as a whole show only a comparatively small change, displaying some decrease in interquartile range but little effect on the overall mean value. At 4% isoflurane exposure, the interquartile range for AVA/AVE signal variance overlaps with the interquartile range for all other neurons.

Figure 8C generalizes this finding across the identified neurons, showing the relationship between baseline (SV_0%_) signal variance and the neuronal activity shift (ΔSV = SV_X%_ - SV_0%_) using linear regression. If the drop in activity of a particular neuron, i.e., its signal variance, in response to isoflurane is proportional to the magnitude of baseline activity, we would expect a linear regression model between SV_0%_ and ΔSV to demonstrate a high value for r^2^ and a negative slope (*β*) that should increase with anesthetic depth – and, indeed, this is what we observe. When performing this regression between the neuronal cohort at 0% isoflurane and that cohort at 2% isoflurane, we find that for AVA/AVE: r^2^ = 0.366, β = −0.449; and for all other neurons: r^2^ = 0.483, β = −0.426. Between the 0 and 4% isoflurane neuronal cohorts, we find for AVA/AVE: r^2^ = 0.743, β = −0.728; and for all other neurons: r^2^ = 0.750, β = −0.642. This finding demonstrates that isoflurane exposure in *C. elegans*, engenders a global suppression of activity across a wide range of neurons, that is proportional to the neuron’s baseline activity level.

## Discussion

Our *C. elegans* neuronal imaging technique measures the activity dynamics of individual identified neurons in a single animal across progressively increasing levels of isoflurane anesthesia. Within the context of the animal’s mapped neuronal connectome, this allows for parsing of neuronal sub-types based on specific cellular characteristics and allows us to determine if key neuronal populations and functional effects exhibit distinct sensitivity to isoflurane. Our system thus allows us to probe the functional effects of volatile anesthetics at the cellular and circuit level within an intact and well-defined nervous system in a way that has not been previously possible.

### Non-specific suppression of neuron activity

Previous studies have suggested a wide range of potential targets for volatile anesthetics including synaptic release, specific post-synaptic receptors or modulation of cellular physiology. We know, from intravenous anesthetics with known targets, that it is possible to elicit an anesthetized state by sufficiently perturbing a single neurotransmitter axis (Brown et al., 2011). We therefore tested if the functional effects of isoflurane anesthesia within an intact nervous system could be attributed to a specific subset of neurons based on these potential targets. However, rather than highlighting a specific neuron-type, our results are remarkable in their uniformity: specific sub-classes of neurons based on neurotransmitter type do not appear dramatically different under isoflurane anesthesia. We see that cholinergic, glutamatergic, and GABAergic neurons all display a similar depression in activity levels with increased isoflurane (Figure 4A). We find that the major excitatory connections in the *C. elegans* nervous system (i.e., cholinergic and glutamatergic synaptic pairs) show similar losses of correlativity under isoflurane that correspond with the general loss of connectivity throughout the nervous system (Figure 4 B, C). Both dopaminergic and GABAergic synaptic pairs show a smaller shift in neuron pair correlativity. Dopaminergic neurons are sensory and may display low baseline activity in our encapsulated assays due to a relative absence of stimuli; this may limit active signaling and neuron pair correlation across all trials. Our inability to detect shifts in GABAergic correlation could be due to the relatively few GABAergic synapses in the *C. elegans* nervous system and consequently a limited sample size. As such, our results are in accordance with over 150 years of anesthetic research which has failed to identify a definitive functional target of volatile anesthetics. Instead, *C. elegans’* simplicity, and the wide-ranging nature of our imaging technique, allows us to clearly demonstrate the effect of isoflurane anesthesia to globally suppress neuronal activity and connectivity across an entire nervous system at the functional level.

We note that our analysis here is limited to the first order physical connectome (i.e., one-to-one synapses). Higher order multi-neuron circuitry as well as extrasynaptic signaling, via neuropeptides, monoamines and other neurotransmitters are known to play an important role in the *C. elegans* nervous system (Gendrel et al., 2016; Bentley et al., 2016; Ripoll-Sanchez et al., 2023; Uzel et al., 2022). Indeed, a functional, rather than anatomic, map of the signal propagation properties of the *C. elegans* nervous system revealed that the functional connectome of the worm can differ quite dramatically from what would be suggested by the physical synapses (Randi et al., 2023). Measurements in this study were also limited to spontaneous activity across the nervous system, rather than stimulus/response sensory-evoked activity. Probing how isoflurane exposure alters patterns of stimulated signal propagation could further inform how the volatile anesthetics generate their systemic effects and reveal what role extrasynaptic signaling may play. Nonetheless, our analysis here supports the assertion that there is no “silver bullet,” i.e., specific neuron or synapse class, that selectively mediates the major functional effects of isoflurane anesthesia in the *C. elegans* nervous system.

Our most striking finding is, in fact, that the isoflurane-induced reduction in neuron activity is proportional to the neuron’s baseline activity in the awake state. This is evident from the linear relationship between the change in activity and baseline activity measured across all neurons (Figure 8C). The effect is dose-dependent, with higher levels of isoflurane resulting in proportionally larger activity suppression. Such results are consistent with a broad-based non-specific mechanism of action such as modulation of neuron physiology, generalized changes in levels of excitatory signaling, or activity-related processes such as cellular metabolism.

### Breakdown of hub-neuron circuitry is a critical component of anesthesia

We find a strong correlation between the in-degree of a neuron and its overall level of suppression in the anesthetized state, both at light and moderate planes of anesthesia. The highest in-degree neurons within the *C. elegans* nervous system belong to a group of highly integrative hub neurons that comprise the locomotory command interneuron circuit (Figure 6B). In particular, we find that the rich club interneurons AVA and AVE, which serve to coordinate forward and reverse movement, exhibit a marked suppression in neuronal activity in response to isoflurane (Figures 7A,B). Correspondingly, these neurons also exhibit the greatest baseline spontaneous activity in the awake state (indicated as red data points, Figure 8).

Isoflurane induced suppression of highly active, highly connected neurons within the *C. elegans* nervous system is consistent with increased system-wide neuron dyssynchrony under anesthesia. Numerous studies have demonstrated the importance of the highly connected hub and command interneurons in controlling and mediating neuronal and behavioral states in *C. elegans* (Atanas et al., 2023; Piggott et al., 2011). Disruption of these neurons impairs the ability of the animal to correctly execute behavioral state transitions. Our previous *C. elegans* neuro-imaging studies demonstrated separately that both the command interneuron circuitry and system-wide behavioral states become disorganized and disrupted under isoflurane anesthesia. However we could draw no conclusions as to the relative importance of specific classes of neurons or neuron types (Awal et al., 2018; Awal et al., 2020). By employing NeuroPAL identification to survey a diverse population of neurons in this study, we show that the activity suppression of the command interneuron circuitry under isoflurane anesthesia is notably larger compared to that of most other neurons in *C. elegans*. Our results therefore inform us about the relative size of the effects of anesthesia across a diverse set of neuron types. Interestingly, Uzel et al. recently demonstrated that acute, selective inhibition of *C. elegans* hub neurons (AVA, AVE, PVC, RIM), via transgenic histamine gated chloride channels, results in a significant suppression of global pairwise neuronal synchrony (Uzel et al., 2022). Thus, active suppression of the same hub interneurons that we have identified here appears to result in a state of system discoordination similar to that of anesthesia (Figure 4B; Awal et al., 2020). Computational network analyses in a simulated mouse neocortical microcircuit suggest that the abolishment of hub neurons disrupts spontaneous network synchrony (Gal et al., 2021) in higher systems as well. Taken together, these results suggest that it is the suppression of highly connected integrator hub neurons that leads to system-wide dyssynchrony under anesthesia.

Although the *C. elegans* nervous system is remarkably simple compared to the mammalian brain, it recapitulates many features of more complex nervous systems including the existence of richly networked and efficiently interconnected hubs (Towlson et al., 2013; Uzel et al., 2022). Many features of nervous systems are scale-invariant (Grosu et al., 2023). The number of external connections to a processing module and the number of connected nodes within that module has been shown to be a spatially scale-invariant property across human brain tract architecture, *C. elegans* microcircuitry, and computer circuits (Bassett et al., 2010). Hubness and rich-club circuitry exist in nervous systems across species and scales, from *C. elegans* to humans (Towlson et al., 2013; Perin et al., 2011; van den Heuvel and Sporns, 2011). Rich-club connections are thought to be critical for efficient communication between network modules but at high wiring cost (measured via physical distance) and metabolic requirements (Liang et al., 2018; van den Heuvel et al., 2012). Interestingly, deficiencies in mitochondrial complex I incur hypersensitivity to volatile anesthetics across the animal kingdom, from *C. elegans* to humans, supporting the importance of metabolically vulnerable neurons in the anesthetic effect (Zimin et al., 2016). Rich-club networks are therefore critical to nervous system function but vulnerable to pathological conditions across neurological disorders such as Alzheimer’s disease and schizophrenia (Griffa and Van den Heuvel, 2018). Correspondingly, we recently drew inference on the importance of the claustrum to the anesthetic effect in humans, given its role as a highly integrating long-range network within the human brain (Bosinski and Connor, 2025). In numerous neuronal activity studies, across species and scales, general anesthesia appears to produce a breakdown in nervous system hub-to-hub communication (Hashmi et al., 2017; Hudetz et al., 2020; Lee et al., 2013; van den Heuvel et al., 2012). Our findings in *C. elegans* provide further evidence of these effects within the simplest of nervous systems, suggesting that neuronal disorganization of the anesthetized state stems from the breakdown of critical, scale invariant hub and rich-club circuitry.

In summary, we measure the activity and connectivity of a large number of individually-identified neurons within the *C. elegans* connectome under increasing levels of isoflurane. This detail in neuronal imaging allows us to probe for cell specific changes in neuronal function under anesthesia. Our results do not identify major neuron-type specific effects on activity or connectivity. Instead, we measure a generalized, proportional suppression of activity dynamics across all identified neurons. Critically, we find that highly active, highly connected hub-interneurons are among the most significantly suppressed. This suppression of critical hub-neurons is consistent with disruption of system-wide neuronal state dynamics and increased neuronal dyssynchrony of the anesthetized state. Thus, while our study does not identify a molecular target for the class of volatile anesthetic agents (i.e., where they act), it illustrates their functional effects on the cellular and circuit level (i.e., how they cause anesthesia through alterations in neuronal and circuit dynamics). As an organism becomes more deeply anesthetized, neuronal activity is uniformly suppressed and communication between the highly active hub elements of the nervous system become disordered. Our results demonstrate how volatile anesthetics can operate to create system desynchrony through generalized effects over a range of scale lengths from individual *C. elegans* neurons to entire human brain regions.

## Data Availability

The raw data supporting the conclusions of this article will be made available by the authors, without undue reservation.

## Supplementary material

Supplementary Figure S1 Discrete survey of neurons identified across recording conditions and animals. Rows represent each neuron class that we deemed to be technically identifiable using NeuroPAL within the region of the head captured by our imaging modality. Columns represent all possible times a neuron could have been identified across 3 recording conditions and 15 animals. A filled box indicates that a particular neuron class was identified in a particular condition in a particular animal. The columns at the far right quantify: the number of animals a neuron class was found in (possible maximum of 15), the number of times a neuron class was found at each recording condition (0%/2%/4% isoflurane), and the number times each neuron class was identified across all recording conditions.
