# Supplementary figures and images for: The volatile anesthetic isoflurane causes global suppression of neuronal activity, disrupting hub neuron function in Caenorhabditis elegans
Source: Front Syst Neurosci. 2026 Jun 12;20:1795887. doi: 10.3389/fnsys.2026.1795887 (PMC13303680; doi:10.3389/fnsys.2026.1795887)

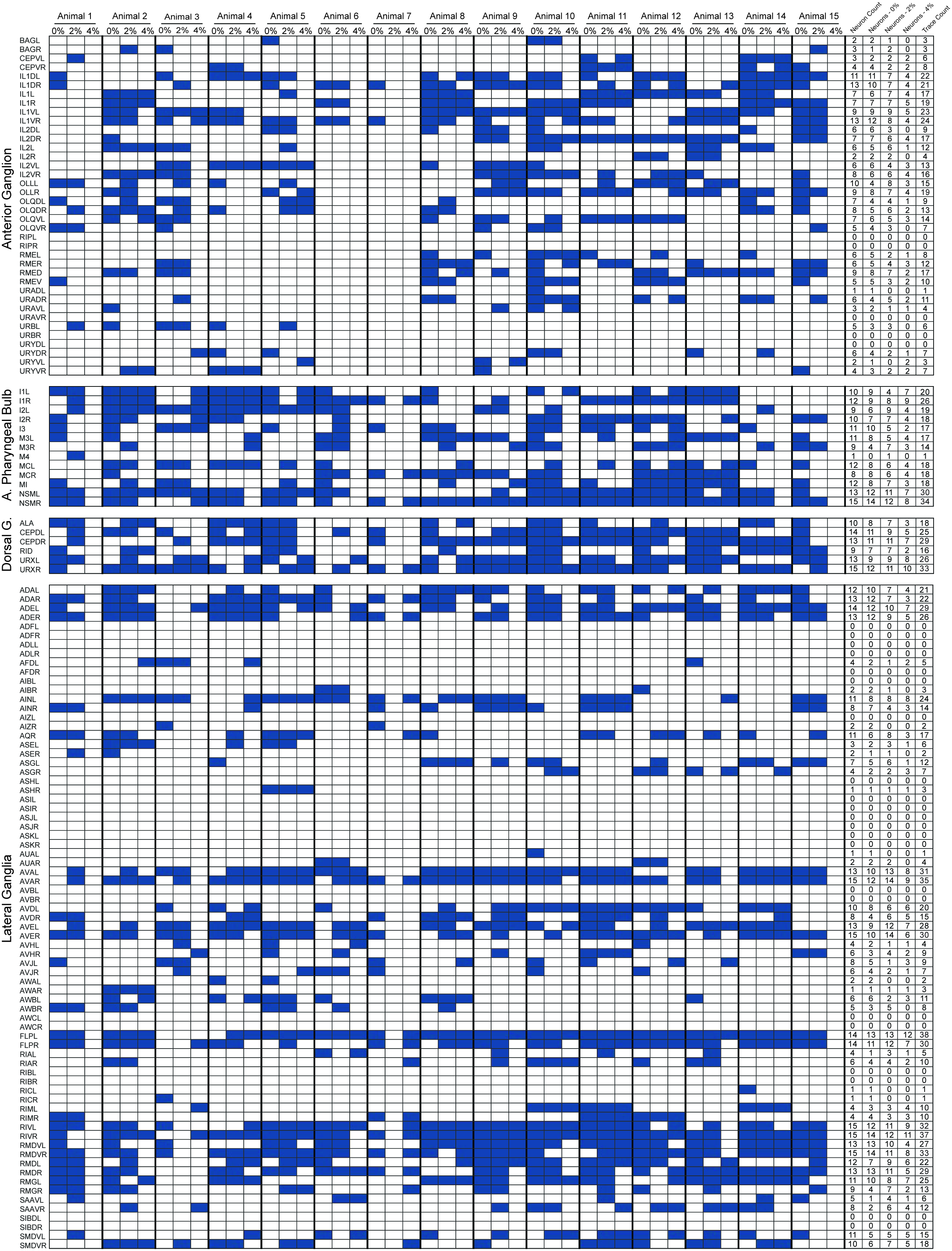

Supplement: Supplementary Figure S1 — Discrete survey of neurons identified across recording conditions and animals. Rows represent each neuron class that we deemed to be technically identifiable using NeuroPAL within the region of the head captured by our imaging modality. Columns represent all possible times a neuron could have been identified across 3 recording conditions and 15 animals. A filled box indicates that a particular neuron class was identified in a particular condition in a particular animal. The columns at the far right quantify: the number of animals a neuron class was found in (possible maximum of 15), the number of times a neuron class was found at each recording condition (0%/2%/4% isoflurane), and the number times each neuron class was identified across all recording conditions. [file Image_1.tif]

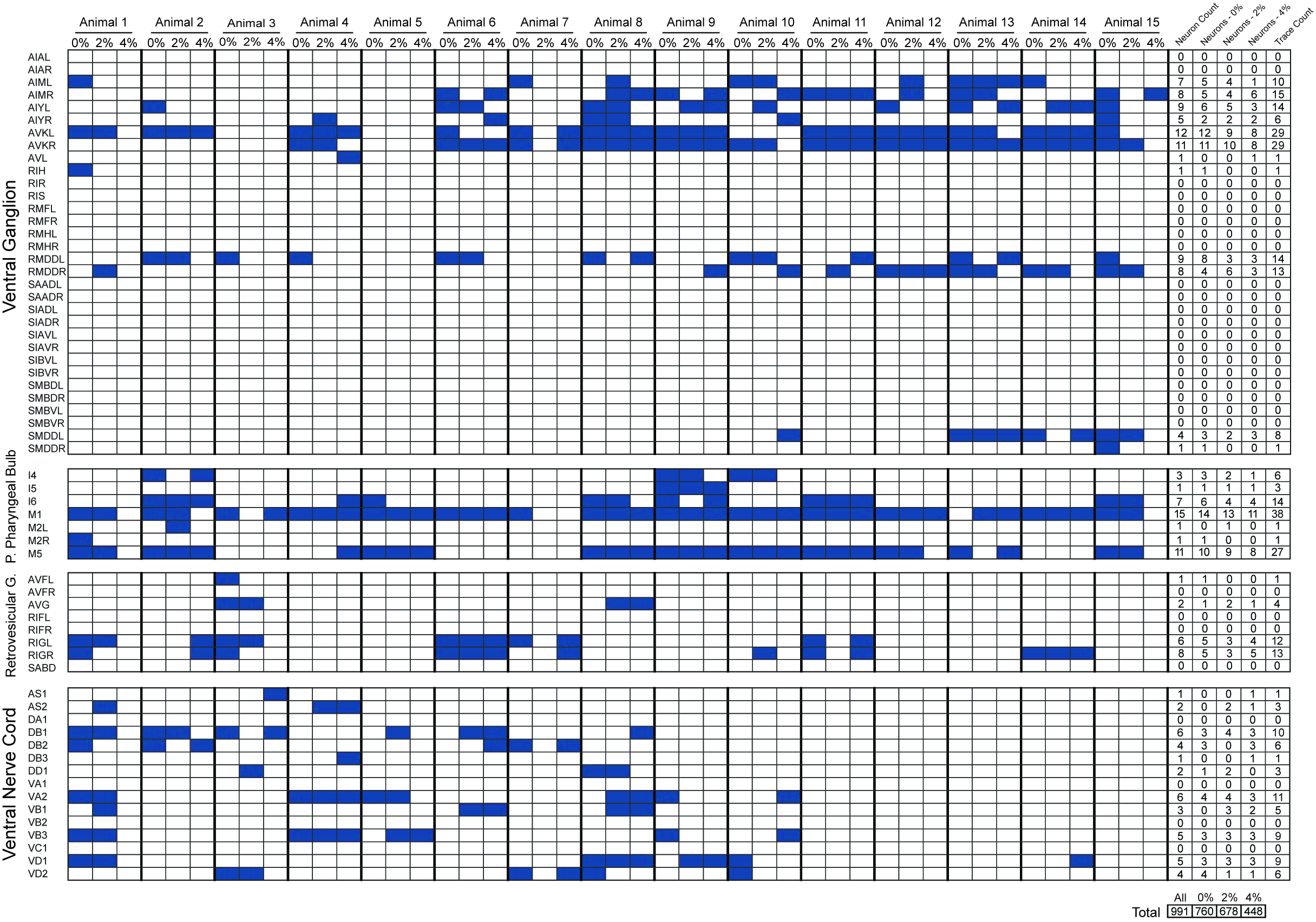

Supplement: Supplementary file 2 [file Image_2.tif]
